# Legal Responsibility of Epileptics

**Published:** 1867-12

**Authors:** 


### Legal Responsibility of Epileptics.

A recent occurrence in Philadelphia has brought up the question of the moral accountability of persons habitually epileptic, in a manner calculated to direct the attention of the medical profession to this important subject with new interest, to lead, we hope, to valuable results. Dr. Isaac Ray, in an article on the subject published in the *American Journal of Insanity* for October, has given an account of the circumstances and discussed the questions involved, in his usual lucid and philosophical manner.

The facts of the case are briefly these. On the third day of May last, George W. Winnemore was convicted in Philadelphia, before the Court of Oyer and Terminer, of the murder, on the 25th of April, of Dorcas Magilton. The unfortunate woman was found by her husband, on returning home after an hour's absence, with her throat cut, quite dead. The prisoner, who was an intimate friend of the family, admitted him to the house, saying that he had just come in, and found her in this condition. The evidence against the prisoner consisted of very few facts. A razor, identified as his, was found in the privy, two bank bills of two dollars each were in the pocket of the deceased the day before, and two bills of two dollars each were found in the prisoner's pocket. Two witnesses who had been looking from their window towards the house for half an hour previously, contradicted his statement that he had come in only a few minutes before. In their defence, the prisoner's counsel contended that it was impossible to identify so common a thing as a razor, of which hundreds of thousands might be made after the same pattern; and that although the bills in his pocket were of the same value as those in the possession of the murdered woman the day before, their exact identity was not established; and, furthermore, that he was not pressed for money, and could easily have obtained it from a brother if he had needed it. The prisoner was shown to have been the victim of epilepsy from two or three years of age until he was ten or eleven years old, sometimes having thirty or forty fits in a day. Full evidence of his having been liable to fits subsequent to boyhood was not obtained until after the trial. The whole trial, however, seems to have been managed without a proper regard for the rights of the prisoner, and the question of his accountability to have been almost wholly ignored. The prisoner was arraigned eight days after the homicide was committed, counsel were assigned to him who had never seen him before, and who knew nothing specially of the case. They were compelled to come to the trial after only two days' preparation, they were obliged to depend on chance for the witnesses that might appear, without any settled plan of defence or opportunity of consulting experts—who, if they had been called in, would have had no chance to examine the condition of the prisoner—much of the medical testimony was excluded, and evidence of the highest importance came too late, although the utmost despatch was used to obtain it. In this way, the evidence of the surgeon of his regiment that he was an epileptic, and was discharged from the service on that account, was excluded, altho' on its way as fast as the United States mail could bring it.

The reason given for hurrying through the trial with such precipitancy was, that the public good required that so foul a crime should meet with speedy retribution. The same spirit prevented a favorable answer to the prayer of the counsel for a new trial, and perhaps influenced the government in their consideration of the report of a committee of medical gentlemen, who visited the prisoner at the request of the counsel, a few days before his execution, and made an examination of his mental condition.

It was in evidence in the course of the trial that the prisoner had always been a quiet, inoffensive, well-disposed young man, who never had been guilty of a crime. He was a firm believer in what is called spiritualism, and was subject to the most extraordinary vagaries, which often put his mother and sister in bodily fear. Twice he had made attempts on his own life, and an uncle had committed suicide.

We are obliged to run over this interesting paper of Dr. Ray's in the most cursory manner, and have no room to refer to many of the extenuating circumstances brought out by him. We cannot forbear, however, to give our readers the document referred to above, testifying to the condition of the prisoner after conviction; it is given in the form of a petition to the Governor:

*To His Excellency, John W. Geary;*

The undersigned, all of whom have been engaged for many years in the care of the insane, have, this day, at the request of Damon Y. Kilgore, had an interview with George W. Winnemore, and in consequonce thereof, beg leave to make the following statement:

Winnemore now, and probably for some time past, shows indications of an abnormal state of mind; of a mental condition which may be attributable to the epileptic fits to which he has been subject from infancy. In regard to its degrees and kind, we feel unable to speak exactly, because one interview, though prolonged to between two and three hours, was not sufficient for the purpose.

We would also state, that epilepsy, especially when of long duration, oftener than otherwise impairs the mental powers, sometimes in one way, sometimes in another, and therefore, whenever an epileptic is charged with crime, nothing less than an exhaustive investigation of his history and of all the circumstances of the case, can remove the suspicion that the crime may have been committed in one of those abnormal conditions that are so often the sequel to epilepsy.

In consideration of these facts, therefore, we respectfully pray your Excellency to stay his execution for a few weeks, in order that a deliberate, scientific investigation of Winnemore's case may be made by the undersigned. ISAAC RAY, M. D.,

Late Supt. Butler Hospital for the Insane of Providence, R. I.

J. H. WORTHINGTON, M. D.,

Supt. Friends' Asylum for the Insane, Philadelphia.

J. PRESTON JONES, M. D.,

Assistant Physician Penn. Hospital for the Insane.

These gentlemen speak of the prisoner as a young man of a quiet, ingenuous manner and cheerful expression. He protested his innocence most emphatically, but expressed himself as willing to die, as he was to be sacrificed for some wise and good purpose known only to God. The prayer of their petition, however, was not granted.

We cannot follow Dr. Ray in all the interesting particulars which he gives of this unfortunate young man's physiological condition' but must be content with a brief extract from his argument upon them, and his statement of the impressions which the facts made upon him:

"Whether from hereditary predisposition or not, it is obvious that the prisoner was born with a nervous system strongly inclined to morbid manifestations. One of these, which actually made its appearance at a very early period, was epilepsy, which of all the forms of cerebral disorder, stands among the gravest. Coincident with this, either as a direct effect, or a collateral result of the original nervous defect, there appeared in childhood instances of unconsciousness, which, pathologically considered, may be affiliated to somnambulism and catalepsy. [A state of mental unconsciousness, not syncope, previously described, which lasted several hours at a time, and in which he did acts which he had no knowledge of afterwards.—Ed.] And these continued to occur through the latter years of his life, though not perhaps in so well-marked a form. It could hardly have been expected that his intellectual operations would entirely escape from the influence of this abnormal condition of the nervous system. Hence his distaste for exact and practical knowledge requiring continuous attention and effort, and his fondness of reverie and dreamy speculation, which needad neither discipline nor preparation. * * * * *

"For legal purposes it might seem necessary to separate the epileptic element from the rest, and ascertain the precise amount of its influence upon the moral character and conduct. But the elements of nervous disorder were too long and too intimately associated together to allow of this. Even under very different circumstances the effect of epilepsy on the mental manifestations is often determined, somewhat, by the training and habits of the individual. Not to the same extent, certainly, that mania is, but enough to be taken into the account in any psychological estimate of its consequences. In this case it may have had the effect of rendering his notions on certain subjects still more extravagant and remote from the line of common belief than they would otherwise have been. Whether or not it ever produced delusions, is a point on which the evidence is not very clear. His spiritualistic experience was that, for the most part, of thousands of other people never supposed to be insane, and yet it is difficult to draw the dividing line between this kind of experience and downright insanity. * * * * * * * * * *

"Spiritualism in any shape is a matter of temperament rather than a deduction of evidence and reason, and thus is furnished an additional proof that Winnemore was endowed with a nervous system peculiarly liable to abnormal activity. I do not mean to convey the idea that the facts of spiritualism are entirely the creation of fancy or of fraud. Many of them are susceptible of proof, and are attested by evidence that places them beyond a reasonable doubt. They indicate the existence of agencies, certainly, that have not been admitted into the philosophy of the schools. It is to be regretted that the prevalent tendency is to ignore them entirely, rather than to make them a subject of scientific investigation. It is surprising that physicians, especially, with such well recognized affections before them as catalepsy, somnambulism, ecstasies and double consciousness, should jump to the conclusion that all the facts of spiritualism and animal magnetism are utterly anomalous and impossible.

"Winnemore's notion about his being a victim, which might seem, at first sight, to be a genuine delusion, was, probably, only a rational notion carried to the utmost verge of extravagance. When his innocence should be proved hereafter to the satisfaction of everybody, as he believed it would, the consequence would be an utter change of popular opinion on the subject of capital punishment, and thus he might regard himself as a sacrifice offered up for the good of humanity—not merely as a martyr whose blood, in the ordinary and regular course of events, would become the seed of a great benefit, but as the favored child of a magnificent destiny prepared and arranged in the councils of the Almighty."

Dr. Ray, in conclusion, seems to incline to the opinion that Winnemore, if guilty, did the act in one of those states of unconsciousness to which he had been subject, but which were not testified to at the trial. He closes his paper as follows:

"In view of what we already know of epilepsy and of what still remains to be learned, we have a right to require the utmost circumspection and the closest investigation whenever the legal liabilities of epileptics are in question. The fact of its existence being established, is it going too far to say that legal responsibility is presumptively annulled, and that the burden of proof lies on the party that alleges the contrary? People are scarcely ready for it yet, perhaps, but to that complexion will they come at last."

We lay this hasty sketch of this most painful case before the profession, in the hope that it may direct their minds most seriously to the important medico-legal questions which it contains. The evidence of the medical men summoned as witnesses seems to have been of the most unsatisfactory character, and worse than useless. Dr. Ray's paper should be read by every one who has the misfortune to be called to testify in such a case.—*Boston Med. Journal.*

